# Evaluation of information presented within mast cell tumour histopathology reports in the United States: 2012–2015

**DOI:** 10.1002/vms3.107

**Published:** 2018-06-07

**Authors:** Jennifer K. Reagan, Laura E. Selmic, Caroline Fallon, Elizabeth A. Driskell, Laura D. Garrett

**Affiliations:** ^1^ Department of Veterinary Clinical Medicine College of Veterinary Medicine Urbana‐Champaign University of Illinois Urbana Illinois; ^2^ Department of Clinical Sciences College of Veterinary Medicine Auburn University Auburn Alabama; ^3^ Department of Pathobiology College of Veterinary Medicine Urbana‐Champaign University of Illinois Urbana Illinois

**Keywords:** Oncology, pathology, small animal, surgical oncology, tumour biology

## Abstract

For canine mast cell tumour (MCT), histopathology reports are one of the main factors considered in the decision‐making process regarding need and type of adjunctive therapy. However, considerable variation exists in types of information reported, especially relating to surgical margins. The purpose of this study was to describe and evaluate how information is presented within canine MCT histopathology reports across the United States. The reports were collected from medical and surgical oncologists from 4 geographic regions of the USA: Midwest, Northeast, South and West. All reports were obtained between January 1st 2012 and May 1st 2015. Inclusion criteria required that the final diagnosis was MCT, a microscopic description was present, and it was not a scar revision. Three hundred and sixty‐eight reports were collected from 26 contributors. While the majority of the reports contained a clinical history (85.9%), information for certain prognostic indicators such as location and mass size was lacking. Grading with both Patnaik and Kiupel systems were described in 76.5% of reports with a single system being used in 7.1% and 15.2% of reports, respectively. Subcutaneous MCT were assigned a grading scheme in 67.2% of reports with 33.3% stating appropriate limitations. Surgical margins were reported in 92% of the reports with 77.2% describing deep and lateral margins separately. Tissue composing the deep margin was only described in 10.9% of the reports. The present results indicate reporting of MCT has variability across pathologists with inconsistencies present in the reporting of clinical history, margin evaluation and subcutaneous MCT grading.

## Introduction

The histopathology report is the primary form of communication between the pathologist and the clinician (Kamstock *et al*. 2011; Newman 2003). The pathologist's ability to provide useful information to the clinician is dependent on the quality of the clinician's input such as providing an accurate history with pertinent prognostic information as well as performing appropriate sample submission and margin marking (Kamstock *et al*. 2011; Brannick *et al*. 2012). Likewise, the pathologist is expected to provide a diagnosis when possible and any histologic parameters that may predict biologic behaviour for that specific tumour type such as reporting of grade (Kamstock *et al*. 2011; Newman 2003).

Kamstock *et al*. (2011) published a consensus providing guidelines and recommendations on veterinary surgical pathology reporting. Generally it was recommended that a report should include the following components: diagnosis, grade when applicable, microscopic description, comments/remarks and references (Kamstock *et al*. 2011). Related to grade, it was recommended that grade be listed after the diagnosis, the features used to arrive at that grade should be described, and a reference should be reported for the grading scheme (Kamstock *et al*. 2011). Other specific features of the report that were discussed included mitotic index (MI) and histologic margins. They stated that MI should be listed as the number of mitotic figures per number of high power field (ideally minimum of 10 high power fields) (Kamstock *et al*. 2011). When reporting margins, the method of trimming should be reported. Also, margin evaluation should include a description of the closest neoplastic cells, an objective measure from the closest neoplastic cell to the margin, and the tissue types/quality composing the margin (Kamstock *et al*. 2011).

Recently the VCS Oncology‐Pathology Working Group MCT Subgroup, (2013) published a consensus on cutaneous mast cell tumour (MCT) grading which recommended that both Kiupel *et al*. (2011) and Patnaik *et al*. (1984) grading systems be reported and that MI should be standardized in a similar fashion as that recommended by Kamstock *et al*. (2011) . It was also stated that grade should be used in conjunction with the overall clinical picture and other prognostic indicators (VCS Oncology‐Pathology Working Group MCT Subgroup, 2013). For MCT, many prognostic factors have been evaluated that may be presented within the histopathology report. Prognostic information that may be included within the history section includes presence of clinical signs (Mullins *et al*. 2006), tumour location (Kiupel *et al*. 2005; Gieger *et al*. 2003; Garrett 2014), number of concurrent tumours (Kiupel *et al*. 2005), stage (Garrett 2014) and tumour size (Mullins *et al*. 2006). While information supplied by the pathologist associated with MCT prognosis includes MI (Garrett 2014; Berlato *et al*. 2015; van Lelyveld *et al*. 2015; Elston *et al*. 2009; Romansik *et al*. 2007), histologic grade (Kiupel *et al*. 2011; Patnaik *et al*. 1984; Garrett 2014; Takeuchi *et al*. 2013; Murphy *et al*. 2004; Stefanello *et al*. 2015; Donnelly *et al*. 2015; Sabattini *et al*. 2015), histologic margins (Mullins *et al*. 2006; Garrett 2014; Donnelly *et al*. 2015; Seguin *et al*. 2006; Schultheiss *et al*. 2011; Scarpa *et al*. 2012; Weisse *et al*. 2002) and various cellular markers (Garrett 2014; Berlato *et al*. 2015; van Lelyveld *et al*. 2015; Takeuchi *et al*. 2013; Seguin *et al*. 2006; Vascellari *et al*. 2013; Maglennon *et al*. 2008; Kandefer‐Gola *et al*. 2015; Scase *et al*. 2006; Costa Casagrande *et al*. 2015; Webster *et al*. 2007).

Histopathology reports are critically important in the clinician's decision‐making process regarding necessity and mode of adjunctive therapy recommended after tumour removal (Kamstock *et al*. 2011; Newman 2003). However, anecdotally, considerable variation exists in the types of information in histopathological reports for MCT especially relating to histologic margins and the grading system used. The purpose of this study was to describe and evaluate the information present within histopathology reports for surgically resected canine cutaneous MCT. We hypothesized that both Kiupel and Patnaik grading systems would be used for cutaneous MCT but not all reports would contain both grades. Also, margin reporting would be variable and not all reports would contain complete information on lateral and deep surgical margins.

## Methods and materials

Histopathology reports for cases diagnosed as MCT from January 1st 2012 to May 31st 2015 were collected via convenience sampling from various clinicians across the United States. The United States were divided into 4 regions (Table 1) based on the regions described in by the United States Census Bureau (USCB, 2017). All clinicians contributing histopathology reports were board certified specialists (medical oncologist or surgeon), however the reports submitted could have been requested by a referring veterinarian or the contributing veterinarian or their colleagues. The institutions associated with the contributing clinicians were comprised of veterinary teaching hospitals and private specialty practices. It was requested that reports pertaining from their most recent MCT cases that fit the inclusion criteria listed below were submitted for evaluation. Contributors were asked to submit up to 15 reports, however, if additional reports were submitted up to 25 were evaluated.

**Table 1:** Distribution of the states by region of the United States of America based on the United States Census Bureau

Region of the United States	States within region
Northeast	ME, NH, VT, NY, MA, CT, RI, NJ, PA, DE, MD, WV, VA
South	KY, NC, TN, SC, GA, AL, MS, FL, TX, OK, AR, LA
Midwest	ND, MN, WS, MI, SD, IA, IL, IN, OH, NE, KS, MO
West	WA, OR, ID, MT, WY, AK, CA, NV, UT, CO, AZ, NM, HI

All clinicians participating in the study were asked to complete a basic questionnaire related to their associated institution and to submit a copy of each final histopathology report. The questionnaire assessed information related to the practice including: type of practice (specialty referral practice or veterinary teaching hospital), the practice's name, contact information and the name/credentials of the clinician collating the reports. Inclusion criteria for the study required the following: submission of a completed questionnaire and final canine histopathology report with diagnosis of MCT, a microscopic description in the histopathology report, and cases submitted were the first attempt at MCT excision. Incisional biopsies were excluded. The reports were evaluated based on recommended general histopathologic reporting guidelines (Kamstock *et al*. 2011), and the types of data evaluated are listed in Table 2. The reports were evaluated by two individuals (JKR and CF). Interobserver error was not assessed, however, JKR reviewed all columns that contained subjective interpretation (see Table 2) and any disagreement was adjudicated by LES.

**Table 2:** Information evaluated within each histopathology report

Report Section	Information evaluated within each section	Answer format
Signalment	Age at the time of the report	Year or missing
	Breed	Breed or missing
	Sex	Sex or missing
Clinical history	Clinical history present	Yes or no
	Number of words^b^	Number or missing
	Adequate history^c^^,^ d	Remaining information evaluated as yes, no or missing if a clinical history was not present
	Age at diagnosis	
	Location	
	Adequate description	
	Number of masses	
	Mass size	
	Mass growth rate	
	Suspected tumour type	
	Method diagnosis	
	Expected surgical margin	
	Marked margins (sutured or inked)^a^^,^ d	
	Current medications	
Gross description	Gross description present	Yes or no
	Tumour size	3D, 2D, 1D or missing
	Location	Yes or no
Microscopic description	Mitotic index	Yes or no
	Number of mitotic figures per HPF	Yes or no
	Tissue of origin^d^	Subcutaneous, cutaneous, or non‐cutaneous
Diagnosis/MCT grading system	Grading system used	Both, Kiupel, Patnaik or none
	If non‐cutaneous/subcutaneous were limitations stated^d^	Yes, no
	Grade given	High/low and/or 1,2,3
Margin evaluation	Margins reported	Yes or no
	Description of neoplastic cells closest to the margin d	Remaining information evaluated as yes, no or missing if margins were not reported
	All margins described^d^	
	Metric measurements used	
	Direction of closest lateral margin^d^	
	Tissue composing the margin^d^	
	Margin tissue quality^d^	
	Trimming method^d^	
	Subjective descriptors used^d^	
	Margins stated complete or incomplete	
Comments	Comments section present	Yes, no
	Additional diagnostics recommend or performed	Yes, no, missing if no comment section
	If applicable the diagnostic recommended was recorded	AgNOR, PCNA, c‐kit IHC, c‐kit PCR, ki‐67, other
	Comments on biologic behaviour	Yes, no, missing if no comment section
	Oncologist consultation recommended	Yes, no, missing if no comment section
	References	Yes, no, NA

a If margins were inked or marked within the gross description this was included within this category.

b Mast cell tumour (MCT) and FNA considered 1 word for count purposes.

c Subjectively adequate history was defined as yes if 3 or more of the specific pieces of information listed within this section below adequate history was present.

d Columns evaluated by both JKR and CF. For information that was considered more subjective in nature reports were evaluated by both evaluators and if a concern arose LES was consulted.

### Statistics

Descriptive statistics were calculated for each category. Continuous variables were assessed for normality by analysing histograms for skewness and kurtosis, and by Shapiro‐Wilk test. If variables were normally distributed mean and standard deviation was presented or if they were not normally distributed the median and range were presented. All statistics were performed using commercially available software^1,2^.

## Results

A total of 395 MCT histopathology reports were received from 26 contributors across the United States. Twenty‐seven reports did not meet the inclusion criteria and were excluded from statistical analysis. Of the remaining 368 reports, 99/368 (26.9%) were from the South, 96/368 (26.1%) were from the Midwest, 91/368 (24.7%) were from the West, and 82/368 (22.3%) were from the Northeast. Laboratories associated with veterinary universities produced 121/368 of the reports (32.9%), private laboratories created 227/368 of the reports (61.7%) and in 20/368 reports (5.4%) the laboratory could not be determined. There were a total of 26 different private companies and universities represented. Pathologist credentials were given on 309/368 reports (83.9%).

### Signalment and clinical history

Information on the patient signalment was present in 316/368 (85.9%) of the reports with 65 breeds represented. The highest frequencies of reports were from the following breeds: Labrador retriever 48/368 (13.1%), mixed breed 45/368 (12.3%), boxer 26/368 (7.1%) and golden retriever 24/368 (6.5%). For sex, 38.3% (141/368 reports) were male, 46.2% (170/368 reports) were female and 15.5% (57/368 reports) were missing this data. A clinical history or description of the lesion was given in 315/368 reports (86.0%). The median number of words in the history was 17 (range 0–317). The description of the clinical history was considered adequate (Table 2) in 175/368 reports (47.6%). The most common historical information provided was the location of the lesion (292/368 reports, 79.3%), suspected tumour type (219/368 reports, 59.5%) and method of diagnosis of the suspected tumour type (122/368 reports, 33.2%). Other information provided included duration of disease (96/368 reports, 26.1%), number of masses present (81/368, 22.0%), size (70/368 reports, 19.0%), growth rate (48/368 reports, 13.0%), age at diagnosis (41/368 reports, 11.1%) and medications the patient had received (31/368, 8.4%).

### Gross and microscopic description

A gross description was reported in 97/328 reports (26.4%) with 1 report containing images of the sample. Of the reports that contained a gross description, 43/97 (44.3%) reported the sample size in three dimensions, 23/97 (23.7%) reported two dimensions and 21/97 (21.6%) reported one dimension. Mitotic index (MI) was given in 126/368 reports (34.2%) and mitotic figures per high power field were listed in 342/368 reports (92.9%). Seven reports (7/368, 1.9%) did not list a MI or mitotic figures per high power field. The tissue of origin was recorded as cutaneous in 280/368 reports (76.1%), subcutaneous in 52/368 reports (14.1%), muscular in 4/368 reports (1.1%), unknown in 4/368 reports (1.1%), submucosal/mucosal in 2/368 reports (0.5%) and the description of the tissue of origin was missing in 26/368 reports (7.1%).

### Histologic margins

Some description of histologic margins was present in 356/368 reports (96.7%), however description of both lateral and deep margins was only present in 284/368 reports (77.2%). The histologic margins were quantified in centimetres or millimetres in 287/368 reports (78.0%). Subjective descriptors (e.g. clean, close, etc.) were used in 88/368 reports (23.9%) while 188/368 reports (51.1%) specifically stated complete vs. incomplete margins. The direction (e.g., cranial, caudal, etc.) of the closest lateral margin was noted in 60/368 reports (16.3%). The tissue type composing the margin, the quality of the tissue composing the margin and a description of the neoplastic cells closest to the margin were rarely recorded (40/368 reports (10.9%), 4/368 reports (1.1%) and 2/368 reports (0.5%), respectively). The method of sample trimming was noted in 62/368 reports (16.8%), and 123/368 (33.4%) of the reports had an indication that the margins were marked with ink or suture. Only 16/368 reports (4.3%) contained the surgeon's planned margin at the time of surgery, which is the surgical margin as opposed to the histologic margin.

### Grading system

A summary of the year of pathology submission and the grading system reported is presented in Table 3. For cutaneous tumours, a grade was given in majority reports with the majority (226/283; 79.9%) reporting both grading schemes. For non‐cutaneous tumours, a grade was stated on 40/50 reports (80.0%), while 29/50 of these reports (58.0%) did not state the limitations of the application of grading systems to these tumour types.

**Table 3:** Grading systems used for reporting the grade of MCT that were cutaneous and non‐cutaneous in origin by year of report submission

Year of evaluation		Both	Kiupel	Patnaik	No grade
2011	Cutaneous^a^			1	
	Non‐cutaneous not stating grading limitations				
	Non‐cutaneous stating grading limitations^b^				
	Unknown				
2012	Cutaneous^a^	5			
	Non‐cutaneous not stating grading limitations	1		6	
	Non‐cutaneous stating grading limitations^b^			1	
	Unknown				1
2013	Cutaneous^a^	25	4	6	
	Non‐cutaneous not stating grading limitations	1		1	
	Non‐cutaneous stating grading limitations^b^	2			
	Unknown				
2014	Cutaneous^a^	152	12	30	
	Non‐cutaneous not stating grading limitations	12	2	4	8
	Non‐cutaneous stating grading limitations^b^	5			
	Unknown				10
2015	Cutaneous^a^	44	3	1	
	Non‐cutaneous not stating grading limitations	2			2
	Non‐cutaneous stating grading limitations^b^	2		1	
	Unknown				1

There were no submission dates visible for 23 reports so these reports have not been included in this table.

a The cutaneous category contains all tumours that were reported as cutaneous in origin or that were not specified and presumed to be cutaneous based on the report.

b For non‐cutaneous mast cell tumour (MCT) no grade should be given, as these grading systems do not apply to non‐cutaneous MCT. These numbers reflect the number of reports that stated this limitation.

### Comments section

Comments were provided in 363/368 reports (98.6%) with 275/368 reports (74.7%) giving general comments on the biologic behaviour of MCT and 8/368 reports (2.2%) giving treatment recommendations. Additional immunohistochemistry (IHC) stains and diagnostics were performed or offered in 131/368 reports (35.6%) with the most common being c‐KIT PCR (124/368 reports, 33.7%), c‐KIT IHC (106/368 reports, 28.8%), Ki‐67 (95/368 reports, 25.8%), AgNOR (72/368 reports, 19.6%) and PCNA (30/368 reports, 8.2%). The percent of reports that offered or gave results from performed additional stains and/or diagnostics for cutaneous MCT were 13.3% (6/45 reports) for grade 1, 38.6% (78/202 reports) for grade 2, 40.5% (15/37 reports) for grade 3, 34.3% (71/207 reports) for low grade and 52.9% (27/51 reports) for high grade. For non‐cutaneous tumours, additional IHC stains and diagnostics were performed or recommended in 20/58 reports (34.5%). A medical oncology consultation was recommended or offered in 71/368 reports (19.3%). References for the comments provided were provided in 296/368 reports (80.4%).

## Discussion

The main purpose of a histopathology report is to convey information from the pathologist to the clinician that is necessary to guide therapy and determine prognosis. Related to MCT, these factors include confirmation of tumour type, histopathologic grade of the MCT, information on the histologic margins and potentially recommendations for further diagnostic tests (Kamstock *et al*. 2011; Newman 2003). This study found that for MCT, while the majority of these factors were presented within the histopathology reports; variation existed in the reporting, especially related to histologic margins and grading of MCT.

The majority of reports (356/368 reports, 96.7%) had a description of histologic margins, however, the level of information conveyed about the histologic margins varied between reports. Almost a quarter of the reports did not describe all margins (i.e. both the lateral and deep histologic margins) and only 60/368 reports (16.3%) described the direction of the closest histologic margin. While omission of this data may be due to lack of thorough evaluation, more likely the failure is related to incomplete reporting, vague wording of the report or an inability of the pathologist to completely evaluate these margins secondary to how the samples were submitted and subsequently processed. An important aspect of histologic margin evaluation lies in the handling of the sample and post‐operative marking of the margins. In this study, 123/368 (33.4%) of the reports had an indication that the margins were marked with ink or suture. While this may underestimate the number of specimens that were marked if this information was omitted from the report, it is likely that many specimens were unmarked. Without marking the borders of the sample, the orientation of the sample in relation to the animal cannot be determined and in some cases it can be difficult for the pathologist to determine the true cut borders or margins. Therefore, histopathology report content depends on both the clinician submitting the sample and the reporting of the pathologist.

Information on all histologic margins, especially the distance of the closest neoplastic cells from the margin, which margins contain the closest tumour cells and the tissue types composing the margin may affect the decision of whether or not to recommend a scar revision. For example, having tumour cells within 1 mm of the margin may not be as concerning if the tissue composing the margin is fascia versus adipose tissue which is generally thought to act as a poor barrier for tumour cell invasion. In this study, the tissue type composing the margin, the quality of tissue composing the margin (e.g. normal, necrotic, thermal damage, etc.) and a description of the neoplastic cells closest to the margin were all rarely recorded (40/368 reports (10.9%), 4/368 reports (1.1%) and 2/368 reports (0.5%), respectively). For MCT, identifying the tumour cells closest to the margin can pose a particular challenge given that clusters of mast cells within tissue or mast cells related to an inflammation can be indistinguishable from neoplastic mast cells (Scarpa *et al*. 2012; Michels *et al*. 2002).

Kamstock *et al*. (2011) recommended reporting histologic margins in objective measures and avoiding use of subjective descriptors such as close or narrow. In this study, the histologic margins were quantified in centimetres or millimetres in 287/368 reports (78.0%) while subjective descriptors were used in 88/368 reports (23.9%). The use of subjective descriptors leaves room for interpretation, as the definition of “close” will vary between individuals. It has also been recommended to specifically state that a histologic margin is complete or incomplete (Kamstock *et al*. 2011), which was stated in 188/368 reports (51.1%). By stating complete versus incomplete histologic margins there is no room for misinterpretation. It can be debated that the distance of the closest tumour cell to the histologic margins is unimportant as the width of the histologic tumour free margin has not been associated with recurrence (Donnelly *et al*. 2015). Therefore, complete versus incomplete excision may be one of the main considerations when advising adjunctive therapy.

The ability of the pathologist to interpret the histologic margins can be affected by the method of post‐operative handing of the sample and the histologic processing. The most common method used for specimen trimming and histologic margin evaluation is the radial method for small or moderately sized masses (Kamstock *et al*. 2011). Using this method, a very small portion of the margin (generally <0.1%)(Becker 2007; Rapini 1990) is actually evaluated; other methods such as tangential sectioning or parallel slicing allow for evaluation of a larger percentage of the margin (Kamstock *et al*. 2011). The radial method also assumes that tumours are symmetric/evenly distributed (Kamstock *et al*. 2011). Understanding which method was used for trimming and therefore the associated limitations is valuable information for the clinician when interpreting histologic margin results in relation to the true cut margins both for their patient as well as for research studies. In this study the method of sample trimming was only reported in 62/368 reports (16.8%).

It was relatively uncommon for a gross description to be present with only 97/368 reports (26.4%) containing this information and only 1 report containing images of the gross sample. The reports were also not standardized in how the dimensions of the sample were reported, with the samples being measured in three dimensions, two dimensions or one dimension. The importance of the gross description is that it can help orient the clinician to the pathologist's perspective. Images or a gross description can be especially useful when the case has been referred and the clinician using the report was not the clinician who initially removed the tumour. In relation to histologic margins, a gross description or image can also help report post‐surgical changes that occur to a specimen such as the degree of translation that has occurred between the skin, subcutaneous tissue and fascial layers, which may affect the pathologist's ability to reliably assess the histologic margins.

For MCT, the biologic behaviour of mucosal, subcutaneous and cutaneous tumours is different (Elliott *et al*. 2016; Newman *et al*. 2007; Thompson *et al*. 2011), which highlights the importance of the pathologist clearly indicating the tumour's suspected origin on the histopathology report within the diagnosis. Overall, the vast majority of reports in this study clearly indicated the origin of the tumour in either the microscopic description and/or diagnosis with description of the tissue of origin only missing in 26/368 reports (7.1%). However, the grading of non‐cutaneous versus cutaneous MCT was far more variable. For cutaneous MCT, a grade was given in the majority of reports while for non‐cutaneous MCT a grade was stated on 40/50 reports (80.0%), 29/50 of these reports (58.0%) did not state the limitations of the application of grading systems to these tumour types.

The primary grading systems used for grading MCT are the Patnaik and Kiupel systems (Kiupel *et al*. 2011; Patnaik *et al*. 1984). These systems were specifically developed for cutaneous MCT and have not been validated for non‐cutaneous MCT (Elliott *et al*. 2016; Thompson *et al*. 2011). In subcutaneous MCT it has been shown that grade is not indicative of behaviour (Thompson *et al*. 2011). Therefore, while 29/50 of these reports (58.0%) gave this as a limitation, the remaining 21 reports used these grading systems and did not state the limitations of these grading systems related to non‐cutaneous MCT. For clinicians that are unaware of this literature, failure to acknowledge these limitations could result in inappropriate monitoring or treatment recommendations for the patient.

For cutaneous MCT it has been recommended that both Patnaik and Kiupel grading systems be reported (VCS Oncology‐Pathology Working Group MCT Subgroup, 2013). In this study, 79.9% of the reports did comply with this recommendation, with the majority of reports being submitted from reports arising from 2014 and 2015. Using both of these systems has been recommend as each system has its strengths/weaknesses and both of these systems have been associated with prognosis in previous papers (Kiupel *et al*. 2011; Patnaik *et al*. 1984; Takeuchi *et al*. 2013; Murphy *et al*. 2004; Stefanello *et al*. 2015; Donnelly *et al*. 2015; Schultheiss *et al*. 2011). For the Patnaik system, the main concerns are (1) that the majority of tumours are intermediate in grade thereby diminishing its prognostic utility and (2) there is a significant amount of interobserver variation due to the subjective nature of the grading system (Sabattini *et al*. 2015; Northrup *et al*. 2005). While the Kiupel system is more objective than the Patnaik system, it has not been evaluated as thoroughly. Recently, studies have been published comparing both grading systems (Takeuchi *et al*. 2013; Stefanello *et al*. 2015; Sabattini *et al*. 2015). Both Sabattini *et al*. (2015) and Takeuchi *et al*. (2013) evaluated the grading systems in relation to survival and both concluded the Kiupel system had superior prognostic value. Stefanello *et al*. (2015) evaluated the grading systems for prognosticating metastatic disease and concluded prognostication should not rely solely on grade but factor in the results of staging as both grade 1, grade 2, and low grade tumours had metastatic disease (5.8%, 16.5% and 14.9%, respectively). However, it was also noted that using both grading schemes showed a difference in metastatic potential as grade 3/high grade tumours metastasized more frequently than grade 2/high grade tumours (49% vs. 15%) (Stefanello *et al*. 2015). Based on the results of this study, continued reporting of both grading schemes is supported.

The history section contains important information for the pathologist as well as clinicians that may subsequently treat the case. The most common historical information provided was the location of the lesion (292 reports, 79.3%), suspected tumour type (219 reports, 59.5%) and method of diagnosis of the suspected tumour type (122 reports, 33.2%). However, the size of the mass was only present in 75 reports (19.8%). Size has been shown to be an important prognostic indicator (Mullins *et al*. 2006) and is useful information for clinicians. If a gross description was present, size may be indicated within that section of the report, however, the size of the mass may be decreased secondary to surgical removal and histopathologic processing (Risselada *et al*. 2015). Generally, it was noted that the history section was truncated; and as interesting side note, the median number of words in the history was 17 (range 0–317). The brief histories supplied on the reports may be due to the history not being reported to the pathologist or may be that the history was shortened or omitted by the pathologist on the report. Clinical patient history that is pertinent to diagnosis or prognosis is important both for the pathologist as well as for future clinicians reviewing the case and is worthwhile information to be included within the submission and histopathology report.

Additional immunohistochemistry stains and/or PCR testing were performed or offered in 131 reports (35.6%) with the most common being c‐KIT PCR (124 reports, 33.7%), c‐KIT IHC (106 reports, 28.8%), Ki‐67 (95 reports, 25.8%), AgNOR (72 reports, 19.6%) and PCNA (30 reports, 8.2%). Subjectively, certain diagnostic laboratories routinely offered these tests suggesting that this is part of their protocol or submission form. Results from immunohistochemistry stains and PCR testing have been associated with prognosis for MCT (Garrett 2014; Berlato *et al*. 2015; van Lelyveld *et al*. 2015; Elston *et al*. 2009; Romansik *et al*. 2007; Takeuchi *et al*. 2013; Seguin *et al*. 2006; Vascellari *et al*. 2013; Maglennon *et al*. 2008; Kandefer‐Gola *et al*. 2015; Scase *et al*. 2006; Costa Casagrande *et al*. 2015; Webster *et al*. 2007), and the use of these tests in conjunction with histopathologic grade may help determine prognosis (Scase *et al*. 2006). The relatively low proportion of reports offering these additional immunohistochemistry stains and diagnostics shows a lack of general consensus on whether this should be included within the report. The discussion of which tests should be recommended or offered and by whom is beyond of the scope of this paper, however, this highlights an area of inconsistency that may be worthy of discussion.

The main limitations of this paper are the subjective nature of evaluation of histopathology reports as well as the difficulty in obtaining a representative population of histopathology reports for the United States. The authors attempted to make the evaluation of the reports as objective as possible by making most categories a question of present versus absent. We also attempted to obtain reports from both academic and private practices from various geographic regions across the United States. However, those chosen to contribute reports were based on knowledge of individuals in the geographic regions and potential willingness to contribute reports, which may have introduced bias. Another source of bias could have been introduced in the assessment of subjective elements of the reports by two different individuals (XXX and XX), potentially introducing interobserver variation in the interpretation of the reports. Since columns that were considered more subjective were evaluated by both individuals, the affects of interobserver are likely small, however interobserver error was not specifically assessed. Also, if the presence of information was slightly questionable, favour was generally given in favour of the information being present, rather than absent. Therefore, any bias would make the results presented within this paper more favourable towards the clinician and pathologist.

The study findings suggested that while histologic margins are generally reported, details about the margins (e.g. direction of the closest margin and tissues composing the margin) and consistency of how histologic margins are reported are generally lacking. While the majority of reports included both the Kiupel and Patnaik grading systems, about a quarter continue to use only one grading system. Also, grade is often reported for noncutaneous MCT without stating the appropriate limitations of using grading systems developed for cutaneous MCT. The histopathology report represents a vital communication between the pathologist and clinician. It is important for clinicians to improve communication with pathologists in the form of improved clinical history reporting and specimen marking. Likewise, this paper highlights the need for discussions on standardization of certain elements of the histopathology report especially in relation to grading and margin reporting.

## Source of funding

The authors had no conflicts of interest or external sources of funding provided.

## Conflict of Interest

The authors have no conflicts of interest to declare.

## Contribution

JKR drafted the manuscript. JKR and CF performed data collection. All the authors read, critically edited and approved the final manuscript.

## Ethics statement

The authors confirm that the ethical policies of the journal, as noted on the journal's author guidelines page, have been adhered to. No ethical approval was required as this is a observational study involving no direct treatment of animals.
